# Molecular Imaging of Inflammatory Disease

**DOI:** 10.3390/biomedicines9020152

**Published:** 2021-02-04

**Authors:** Meredith A. Jones, William M. MacCuaig, Alex N. Frickenstein, Seda Camalan, Metin N. Gurcan, Jennifer Holter-Chakrabarty, Katherine T. Morris, Molly W. McNally, Kristina K. Booth, Steven Carter, William E. Grizzle, Lacey R. McNally

**Affiliations:** 1 Stephenson School of Biomedical Engineering, University of Oklahoma, Norman, OK 73019, USA; Meredith.jones@ou.edu (M.A.J.); bmaccuaig9@gmail.com (W.M.M.); africk256@ou.edu (A.N.F.); 2 Stephenson Cancer Center, University of Oklahoma, Oklahoma City, OK 73104, USA; jholter2@ouhsc.edu (J.H.-C.); katherine-morris@ouhsc.edu (K.T.M.); mwmcnally@hotmail.com (M.W.M.); Kristina-Booth@ouhsc.edu (K.K.B.); Steven-Carter@ouhsc.edu (S.C.); 3 Department of Internal Medicine, Wake Forest Baptist Health, Winston-Salem, NC 27157, USA; scamalan@wakehealth.edu (S.C.); mgurcan@wakehealth.edu (M.N.G.); 4 Department of Medicine, University of Oklahoma, Oklahoma City, OK 73104, USA; 5 Department of Surgery, University of Oklahoma, Oklahoma City, OK 73104, USA; 6 Department of Pathology, University of Alabama at Birmingham, Birmingham, AL 35294, USA; wgrizzle2@gmail.com

**Keywords:** molecular imaging, inflammation, cardiovascular disease, rheumatoid arthritis, chronic obstructive pulmonary disease, graft vs. host disease, image analysis, machine learning

## Abstract

Inflammatory diseases include a wide variety of highly prevalent conditions with high mortality rates in severe cases ranging from cardiovascular disease, to rheumatoid arthritis, to chronic obstructive pulmonary disease, to graft vs. host disease, to a number of gastrointestinal disorders. Many diseases that are not considered inflammatory per se are associated with varying levels of inflammation. Imaging of the immune system and inflammatory response is of interest as it can give insight into disease progression and severity. Clinical imaging technologies such as computed tomography (CT) and magnetic resonance imaging (MRI) are traditionally limited to the visualization of anatomical information; then, the presence or absence of an inflammatory state must be inferred from the structural abnormalities. Improvement in available contrast agents has made it possible to obtain functional information as well as anatomical. In vivo imaging of inflammation ultimately facilitates an improved accuracy of diagnostics and monitoring of patients to allow for better patient care. Highly specific molecular imaging of inflammatory biomarkers allows for earlier diagnosis to prevent irreversible damage. Advancements in imaging instruments, targeted tracers, and contrast agents represent a rapidly growing area of preclinical research with the hopes of quick translation to the clinic.

## 1. Introduction

Inflammation can manifest in all areas of the body and is often the common denominator between a plethora of diseases and infections. There is a current upsurge in preclinical and translation research to uncover the exact role that inflammation may have in disease progression to make a more accurate diagnosis. Much of this research focuses on imaging inflammation by targeting the immune system. When a pathogen elicits an immune response, there is an upregulation of immune cells such as macrophages, monocytes, and lymphocytes [1]. Monocytes and macrophages are recruited to the infection site where they proliferate and phagocytose the pathogen; it is through this phagocytotic mechanism that exogenous imaging agents can be internalized and the inflammatory response can be imaged [2]. Imaging of lymphocytes is mainly done through radiolabeled antibodies [3,4,5,6]. Molecular imaging of these cells allows for the non-invasive, in vivo visualization of these immune cells to characterize the extent and severity of the disease. Visualization of these immune cells as inflammatory biomarkers will have significant effects on the fields of personalized medicine and early diagnostics of inflammatory disease.

Molecular imaging relies on the presence of endogenous or exogenous contrast agents for the identification of inflamed tissue. While reliance on endogenous contrast is of interest due to decreased risk to patients, lack of specificity in regard to molecular processes limits the accuracy and application of such agents such as hemoglobin and deoxyhemoglobin [7,8]. As a result, molecular imaging has traditionally required tracer molecules specific for biological processes. These tracers consist of a contrast-generating agent, e.g., fluorescent dye, which is targeted for a molecule/function within the body. Such tracers offer high, controllable, and specific contrast, which is unachievable with endogenous contrast alone. Tracers have been utilized across many modalities; examples include radioactive atoms applied to sugars for metabolism tracking with positron emission tomography (PET)/SPECT [9], iodine-labeled tracers for X-ray-based imaging [10], and lanthanides that respond to an external magnetic field for MRI [11]. However, further development of small molecule tracers has slowed significantly due to toxicity concerns and poor sensitivity as a result of weak signal specificity and rapid bodily clearance [1]. The utilization of nanoparticles is a potentially viable method to add exogenous contrast for the purpose of molecular imaging in efforts to overcome limitations that often plague fluorescent probes. While many types of nanoparticles have shown potential, nanoparticles consistently miss expectations in a clinical setting, owing to poor target specificity. Recent advancements in active targeting have improved such outlooks [12]. Approaches to functionalize nanoparticles provide increased specificity through targeting extracellular receptors or key features of the target environment. Identification and exploitation of the molecular signature of diseases allows for the development of novel imaging probes to reveal pathological information about the tissue without the need for invasive biopsies.

Standard clinical imaging techniques such as computed tomography (CT), magnetic resonance imaging (MRI), and ultrasound (US) are traditionally used to reveal anatomical information, providing information required for diagnosis but do not yield molecular information that could be critical for identifying appropriate treatments. For example, the monitoring of response of tumors to therapy uses the Response Evaluation Criteria in Solid Tumors (RECIST) score, which is reliant on changes in tumor size [13]. It may take multiple weeks for a measurable change in tumor size to be observed; however, molecular changes will precede anatomical changes. Recent advancements in contrast agents allows for the extraction of functional and molecular information as well as anatomical information from standard imaging modalities.

The choice of contrast agent for MRI is dependent on the objective of the imaging session. T1-weighted MRI involves the injection of a paramagnetic metal agent, often gadolinium based, which shortens the T1 relaxation time resulting in an enhanced signal [14]. The low sensitivity and limited specificity of gadolinium-based contrast agents renders T1-weighted MRI suboptimal for molecular imaging [15]. T2-weighted MRI most often involves the injection of superparamagnetic iron oxide nanoparticles (SPIONs), resulting in negative contrast enhancement. SPIONs usually range in size from 10 to 100 nm and are often dextran-coated to increase biocompatibility [16,17]. The uptake of SPIONs by active macrophages at sites of inflammation make T2-weighted MRI an appropriate choice for molecular imaging of inflammation [16,18]. MR spectroscopy is another MR technique that acquires the molecular spectra of the tissue of interest in order to reveal information about the concentration and presence of different metabolites in the tissue [19]. While MR spectroscopy provides more information than standard MRI, it is limited by poor spatial resolution and low sensitivity, since the agents of interest exist in very low concentrations [20]. Chemical exchange saturation transfer (CEST) MR imaging involves the exchange of magnetization from the target agent to the surrounding water molecules so that the signal reduction through the saturation effect is seen only on the water molecules and not on the target agent. CEST imaging is dependent on the chemical composition of the target metabolite and the radiofrequency pulse that initiates the chemical exchange of the proton [20]. Then, the sensitivity of CEST imaging is directly related to the chemical exchange rate of the proton transfer, allowing for molecular imaging of specific metabolites that are found at low concentrations [21]. While the majority of research investigating CEST agents is primarily focused on oncology [22], there has been preclinical work investigating the role of CEST imaging and MR spectroscopy in neuroinflammation [23,24,25].

As inflammation is a hallmark of many diseases, the ability to image inflammatory biomarkers on the molecular level will allow for better understanding of disease pathophysiology and ultimately better patient care. The purpose of this review is to highlight the existing molecular imaging techniques used to assess the inflammatory state of cardiovascular disease, rheumatoid arthritis, chronic obstructive lung disease, and gastrointestinal disorders.

## 2. Imaging Inflammatory Disease

### 2.1. Cardiovascular Disease (CVD)

Cardiovascular disease (CVD) is the leading cause of death worldwide [26,27]. CVD is a broad term that encompasses many heart and circulatory system conditions, most of which develop gradually and are only diagnosed after the presentation of symptoms, which often result in fatality, mainly heart attack or stroke [28]. One person will die every 36 s from CVD in the United States alone, and with an increase in the number of smokers and growing obesity rates—two major risk factors for developing CVD—it is now more important than ever to focus on the development of early screening tools to identify the markers of CVD before it is too late [29,30,31].

Atherosclerosis occurs when plaque builds up inside the artery; over time, this plaque will harden and the artery will narrow, limiting blood flow, which often results in cardiovascular disease [32]. This plaque buildup is often only detected at the onset of symptoms, such as myocardial infarction or stroke, which are two of the most common causes of mortality in the United States and Europe [31]. At present, catheter-based X-ray angiography or intravascular ultrasound is used to identify coronary atherosclerosis, but this procedure is extremely invasive and only yields anatomical information about the degree of stenosis [26,33,34]. Non-invasive molecular imaging techniques must be utilized to characterize the plaque activity to determine which patients are extremely high-risk and require immediate intervention. Coronary CT angiography (CCTA) is a method for identifying the degree of stenosis and the plaque composition [35]. CCTA is able to score the degree of calcification of the coronary plaque, which is a strong predictor of a serious cardiovascular event [36,37]. While CCTA does provide functional information about CVD, it falls short of being a true molecular imaging technique, as it does not visualize changes on molecular level.

**Figure 1:**
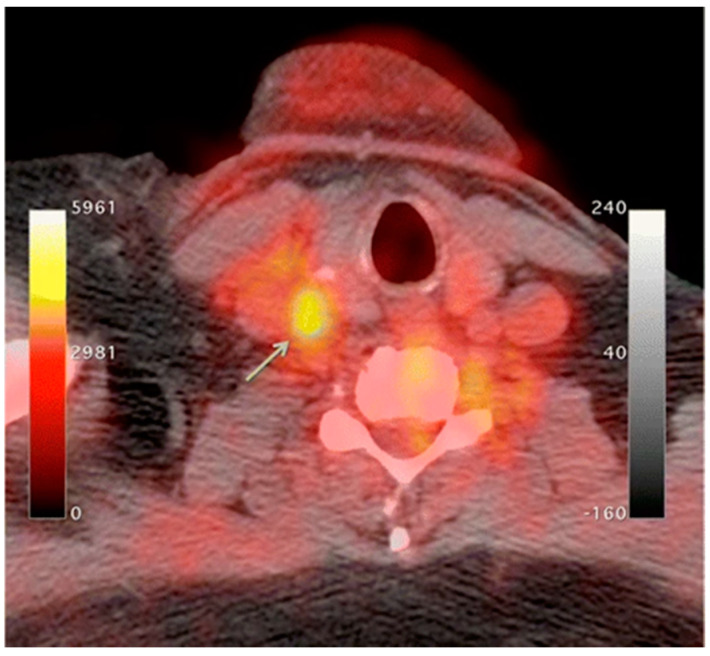
^18^F-Flourodeoxyglucose (^18^F-FDG) positron emission tomography (PET)/computed tomography (CT) imaging of activated macrophages to visualize vulnerable plaques through increase in glucose metabolism. Higher ^18^F-FDG update is seen in the right common carotid artery (arrow) [47].

An increase in macrophage activity, reflective of inflammation, has been linked to a higher risk of plaque rupture; therefore, molecular imaging of macrophage activity in the arteries can help identify areas where plaque may be building [38,39,40]. ^18^F-Flourodeoxyglucose (FDG) PET imaging is commonly used to image the inflammatory component of atherosclerosis [41,42,43,44]. ^18^F-FDG is a radiolabeled glucose molecule, which is internalized by cells through the same mechanism in which glucose is metabolized. Both ^18^F-FDG and glucose are phosphorylated by hexokinase, where ^18^F-FDG becomes ^18^F-FDG-6-phosphate and glucose become glucose-6-phosphate. ^18^F-FDG-6-phosphate cannot be further metabolized by glucose-6-phosphate isomerase; therefore, it remains inside the cell for PET imaging [45]. In atherosclerosis, the accumulation of macrophages at locations of active plaque buildup requires a large amount of glucose, thus causing the upregulation of glucose transporters on the surface of these macrophages. Therefore, increased ^18^F-FDG uptake will be seen at locations of increased macrophage density, which is reflective of inflammation and active plaque buildup (Figure 1) [38,46]. It is unknown what the influence of ^18^F-FDG uptake from other cells, such as, neutrophils, endothelial cells, and lymphocytes, has on the observed signal [33,40]. Once the plaque cells have calcified, ^18^F-FDG uptake will subside substantially, making this type of PET imaging ineffective. PET imaging of atherosclerosis using ^18^F-FDG requires a circulation time of 2–3 h to allow for accumulation in the arterial wall and the decay or excretion of background levels of ^18^F-FDG [40]. ^18^F-FDG PET imaging in oncology typically needs 1 h of circulation time before imaging can begin.

^18^F-FDG PET imaging is non-specific; therefore, it is complicated by highly metabolic neighboring tissues such as myocardial cells and neurons [38,47,48]. The suppression of myocardial ^18^F-FDG uptake can be achieved through dietary manipulation (high-fat, low-carb) to shift the body into beta-oxidation of fatty acids instead of metabolizing glucose as a primary energy source to try and limit this background activity [49,50]. Other radiotracers can be utilized that are specific to macrophages, limiting the effects from other highly metabolic cells. Translocator protein (TSPO)/peripheral benzodiazepine (PBR) receptors are overexpressed in activated macrophages, which is a great option for active targeting [51]. ^11^C-PK11195, (1-(2-Chlorophenyl)-N-(11C)methyl-N- (1-methylpropyl) -3-isoquinoline carboxamide) is a radiolabeled TSPO ligand that has been used as a PET tracer to visualize inflammatory plaque in atherosclerosis [51,52,53,54]. ^11^C-PK11195 uptake in patients with atherosclerosis was higher in patients who had a myocardial infarction or stroke compared to patients who were asymptomatic [53]. Other radiolabeled TSPO targeted ligands include ^18^F-GE-180, which showed a better signal-to-noise ratio and lower non-specific binding; more work must be done to validate this radiotracer [55].

^68^Ga-DOTATATE is another radiolabeled tracer that can be used to target inflammatory plaque in atherosclerosis by targeting the somatostatin receptor subtype 2 (SSR-2), which is also overexpressed on activated macrophages [56,57]. A copper radiolabel (^64^Cu) is frequently substituted for gallium because of the longer half-life and shorter positron range, which allow for better spatial resolution [47,58]. CXC-motif chemokine receptor 4 (CXCR-4) is also overexpressed on many immune cells, particularly monocytes and macrophages, making this receptor a good target for imaging inflammatory plaques of atherosclerosis [59]. Radiolabeled pentixafor, ^68^Ga-pentixafor, targets this CXCR-4 receptor for the quantification of arterial inflammation in atherosclerotic plaques [59,60,61].

As plaque builds up inside the artery, macrophages become active, and the region often becomes hypoxic due to the reduced oxygen diffusion efficiency from the thickening of the vessel wall. As active macrophages reflect sites of inflammation, it is suspected that macrophage activity is partially mediated by hypoxia as atherosclerotic plaques overexpress hypoxia-inducible factor 1-alpha (HIF-1α) [33,47,62]. There is ongoing research that focuses on the imaging of hypoxia as a surrogate biomarker of plaque inflammation and atherosclerosis. Radiolabeled ligands such as ^18^F-fluoromisonidazole (FMISO) or ^18^F-EF5 have been used to detect atherosclerotic plaques through PET imaging of hypoxia preclinically; more work must be done to advance these findings to the clinic [63,64,65].

### 2.2. Rheumatoid Arthritis

Rheumatoid arthritis (RA) is an autoimmune disorder that is characterized by chronic inflammation of the joints often causing degradation of the cartilage and bone, leading to a diminished quality of life due to musculoskeletal deficits and chronic pain [66]. For every 1000 adults, five will have RA, making it one of the most prevalent chronic inflammatory conditions worldwide [67]. RA etiology is not exactly known due to the synergistic effects of epigenetics [68] and environmental factors (smoking [69,70], obesity [71,72,73], and alcohol consumption [74,75,76]). Autoantibodies such as antibodies to citrullinated protein antigens (ACPAs) or rheumatoid factor (RF) have well-established roles in RA as accurate predictors of disease severity [77,78,79]. The current standard of care for the diagnosis of RA is through blood work to monitor the erythrocyte sedimentation rate (ESR), C-reactive protein levels (CRP), RF, and ACPAs [77,80] or anatomical imaging through MRI and ultrasound [81]. Power Doppler ultrasound (PDUS) is an US technique that is commonly used in the evaluation of RA, as it can visualize blood flow as well as anatomical information. The locations of active inflammation will have increased blood flow, making PDUS a good choice for not only diagnosing RA but also for assessing the severity and response to treatment [82,83].

Synovial membrane inflammation (synovitis) is a key characteristic of RA that involves the upregulation of both innate and adaptive immune cells and fibroblast-like synoviocytes (FLS) [84]. This immune response coupled with FLS results in inflammation and the activation of osteoclasts that leads to the degradation of cartilage [85,86]. The synovial fluid contains a variety of activated macrophages, B cells, and T cells, all of which are good targets for the molecular imaging of RA. The overexpression of inflammatory biomarkers can damage the existing vasculature, resulting in the enhanced permeability and retention (EPR) effect [87]. The newly permeable environment allows for the passive targeting of the immune cells of an inflammatory response. SPION-based contrast agents are small enough to penetrate the synovial fluid where they are phagocytized by active macrophages and can be visualized by T2-weighted MRI [18,88,89].

Activated macrophages can also be imaged using ^18^F-FDG PET imaging in the same manner described above [90,91,92]. While ^18^F-FDG PET imaging targets activated macrophages through elevated levels of glucose metabolism, there are more specific methods used to image active macrophages in RA. Folate receptor β (FRβ), a glycosylphosphatidyl plasma membrane anchored protein used to internalize folate needed for DNA synthesis and cell division, is overexpressed on activated macrophages in the synovial fluid, making it an attractive target for the molecular imaging of RA [93,94]. Radiolabeled folic acid can be imaged through scintigraphy or PET imaging for the detection of inflammation in the joints (Table 1) [93,95,96,97]. Spatial resolution of PET images is poor; a fluorescently labeled folate probe (NIR2-folate) can be visualized with NIR fluorescence imaging with greater spatial resolution, but this technique is limited by penetration depth due to light scattering in tissue [98]. Many other methods exist for targeting activated macrophages in RA [99].

**Figure 2:**
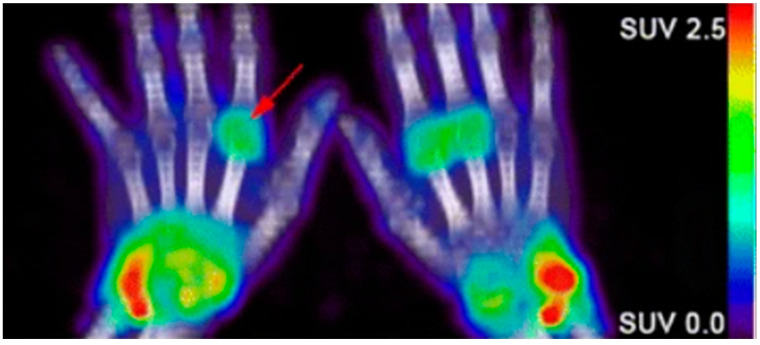
Confirmation of rheumatoid arthritis (RA) in the wrists/hands of patients using ^89^Zr-rituximab PET imaging to target B-cell accumulation [105].

Due to the abundance of immune cells in the synovial fluid, there is an overexpression of inflammatory cytokines that elicit certain cellular responses that can then be targeted for imaging. The presence of interleukin-1 and tumor necrosis factor alpha (TNF-α) stimulate the transient expression of surface protein E-selectin on vascular endothelial cells and the overexpression of matrix metalloproteases (MMPs) in the synovial fluid. Anti E-selectin antibodies and MMP-targeted probes can be either radiolabeled or conjugated to an NIR dye and visualized through scintillation/PET or NIR fluorescence imaging [100,101,102,103,104]. Biologicals used as therapeutics for RA can also be radiolabeled and used to image RA. Rituximab, a monoclonal antibody that targets CD20, a cell surface marker that is expressed on most B cells, can be radiolabeled and used as a probe for the in vivo molecular imaging of RA based on B lymphocyte accumulation in the synovial fluid (Figure 2) [4,105,106]. Infliximab, a monoclonal antibody that targets tumor necrosis factor alpha (TNFα), has also been radiolabeled with ^99m^Tc, which demonstrated a superior sensitivity to inflammation than MRI and clinical examinations in patients with RA [107,108,109].

Carbohydrate-binding proteins, L-selection and P-selection, are involved in the movement of immune cells before and during the inflammatory response [110]. Polyanionic dendritic polyglycerol sulfate (dPGS) targets inflammation through binding with these selectins. Conjugation with indocyanine green (ICG), an NIR fluorescent dye, allowed for the in vivo differentiation of RA-positive joints from RA negative joints in a preclinical rat arthritis model as seen by a 3.5-fold greater fluorescence imaging signal [111]. As the clinical translation of NIR fluorescence is limited by low penetration depth, multispectral optoacoustic tomography (MSOT) can overcome those limitations. MSOT imaging is based on a light-in, sound-out approach, having all the benefits of optical imaging but allowing for increased depth penetration, since photon scattering is irrelevant to acoustic waves [112]. Then, NIR-labeled dPGS can be imaged at much greater depths using MSOT [113].

### 2.3. Chronic Obstructive Pulmonary Disease (COPD)

Chronic obstructive pulmonary disease (COPD) is a preventable, but underdiagnosed inflammatory disease with an extremely high morbidity and mortality rate [114]. Approximately 90% of all COPD cases are related to smoking, yet only 20% of smokers will develop COPD, suggesting that other environmental and genetic factors must also play a role [115,116]. COPD is characterized by airway obstruction due to chronic inflammation and tissue damage caused by a decrease in alveolar elasticity and gas exchange, which ultimately leads to an irreversible decrease in lung function [117]. Pulmonary function testing (PFT) to measure airflow coupled with conventional imaging modalities, CT or MRI, to visualize morphological changes in the airway, is the current standard for diagnosing COPD [118]. Since COPD is an inflammatory disease, these imaging modalities must infer about the inflammatory state through surrogate biomarkers such as airway thickness and airway wall area [117]. Emphysema and chronic bronchitis are two subtypes of COPD that have very distinct molecular characteristics. Emphysema is an irreversible condition induced by smoking or inhaling irritants that destroys the alveoli; this leads to a decrease in the surface area of the lungs, making it difficult to obtain oxygen, causing inflammation of the lung parenchyma [119,120]. Chronic bronchitis is the persistent inflammation of the bronchial tubes due to a chronic cough, which leads to sputum build up in the airways, restricting airflow [121,122]. Early identification of COPD and proper differentiation of different phenotypes is imperative for the development of a proper treatment plan.

Molecular imaging techniques have been developed to target the inflammatory response of COPD. As the airways become inflamed, there will be changes in the pulmonary blood flow as well as airflow. These changes often precede morphological changes that can be detected by CT. Perfusion scintigraphy through the injection of ^99m^Tc-labeled macroaggregated albumin coupled with ventilation scintigraphy through the inhalation of either an inert radioactive gas (^81m^Kr or ^133^Xe), an aerosol-based ^99m^Tc-labeled DTPA, or Technegas (^99m^Tc-labeled carbon particles) will uncover aspects of the heterogeneity of the disease that cannot be seen using PFT or CT [123]. A great comparative study of these radiolabeled tracers in ventilation scintigraphy is found here [124]. The Ventilation to Perfusion (V/Q) ratio obtained will yield important information about regional differences in airflow and inflammation, where larger V/Q values indicate emphysema and lower values reflect chronic bronchitis [125,126]. Similarly, MRI using hyperpolarized noble gas (^3^He or ^129^Xe) can also be used to assess the ventilation status through imaging of the airspaces of the lungs rather than the tissue [127]. Apparent diffusion coefficient (ADC) maps of the hyperpolarized gas can be obtained on a voxel-wise basis using diffusion-weighted MRI (DWI-MRI), where high ADC values reflect areas of severe disease [128,129]. While hyperpolarized MRI is able to visualize the ventilation deficiencies associated with COPD, it is limited by spatial resolution and the ability of the patient to hold their breath.

**Figure 3:**
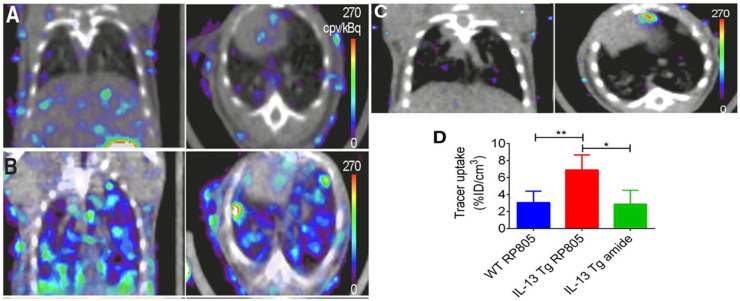
Coronal (left) and transversal (right) SPECT/CT imaging of matrix metalloproteases (MMPs). (**A**) Wild-type mice injected with ^99m^Tc-labeled RP805 (**B**) IL-13 transgenic mice injected with ^99m^Tc-labeled RP805 (**C**) IL-13 transgenic mice injected with an amide analog tracer as a control. (**D**) Quantification of uptake in SPECT images. * *p* < 0.01 ** *p* < 0.001 [136].

As with other inflammatory diseases, COPD can also be visualized through immune cells. ^18^F-FDG PET/CT imaging is commonly used to monitor the metabolic activity of immune cells to diagnose and identify disease severity [130,131,132,133]. Since ^18^F-FDG is a non-specific biomarker of immune activity, the addition of ^11^C-PK11195, a macrophage-targeted radiotracer, allows for the non-specific visualization of neutrophil activity as well as the more specific visualization of macrophage accumulation. A study involving six patients with COPD and five control subjects saw a greater accumulation of ^18^F-FDG in all COPD patients compared to control, and greater ^11^C-PK11195 accumulation in four of six COPD patients compared to control [134]. Macrophages will secrete matrix metalloproteases (MMPs) and many other cytokines, which are all attractive options for the molecular imaging of COPD. Using a mouse model of COPD, a radiofluorinated probe, ^18^F-IPFP, was developed and tested to target MMP-9 and MMP-12; the accumulation of ^18^F-IPFP was 4× higher in the lungs of COPD mice than in normal mice [135]. ^99m^Tc-labeled RP805 is another MMP targeted radiotracer that saw significantly greater accumulation in IL-13 transgenic mice than control mice using SPECT/CT (Figure 3) [136].

### 2.4. Gastrointestinal

Different gastrointestinal (GI) diseases can present with common, non-specific symptoms such as diarrhea and abdominal pain, making accurate diagnosis challenging without molecular information in addition to history and physical exam. [137,138]. Globally, the prevalence of inflammatory GI conditions such as inflammatory bowel disease (IBD) has increased significantly over time [139], particularly in developing countries [140]. Several causes, including genetic factors, diet, and infection, can result in inflammation of the GI tract. Identification of GI inflammation can aid in monitoring response to interventions. Subsequently, appropriate treatment can be administered to relieve symptoms or prevent disease progression. This can be especially critical in lowering patient risk for colorectal cancers [141].

**Figure 4:**
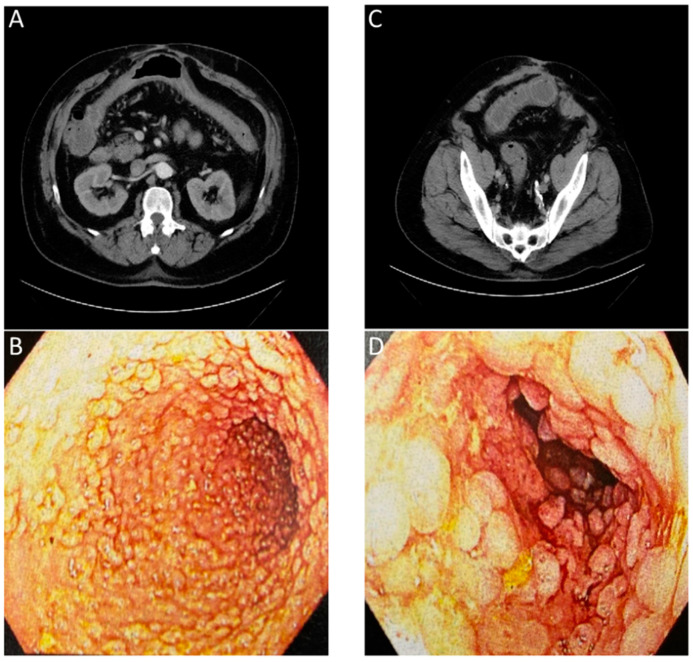
Images of a patient with history of chronic diarrhea that is occasionally bloody. CT ordered for unrelated reasons incidentally showed non-specific inflammation. Correlation with endoscopy showed substantial chronic inflammation. (**A**) CT with arrows showing inflammation of transverse colon. (**B**) Endoscopic images of transverse colon with diffuse pseudopolyps. (**C**) CT with arrows showing inflammation of sigmoid colon. (**D**) Endoscopic images of sigmoid colon with diffuse pseudopolyps. The lack of an inflammation or cancer specific contrast agent for the CT or endoscopic evaluation required a biopsy to confirm a lack of neoplasia.

Historically, tests using blood, stool, or biopsied tissue samples have been paired with invasive imaging techniques, such as endoscopy, to diagnose and assess patient GI disease [141]. Currently available invasive and non-invasive imaging techniques such as endoscopy, CT, MRI, and US, can show the macroscopic structural abnormalities associated with inflammatory bowel disease such as bowel wall thickening, abscesses, or fistulas to identify the scope of disease [142]. When combining multiple standard imaging modalities, the presence of inter-clinician reader variability and the lack of molecular information contained in the images (Figure 4) often requires a biopsy for an accurate diagnosis. In the context of Figure 4, the numerous lesions within the colon result in a higher potential of a biopsy sampling error and the possibility to miss areas of early-stage colon cancer.

**Figure 5:**
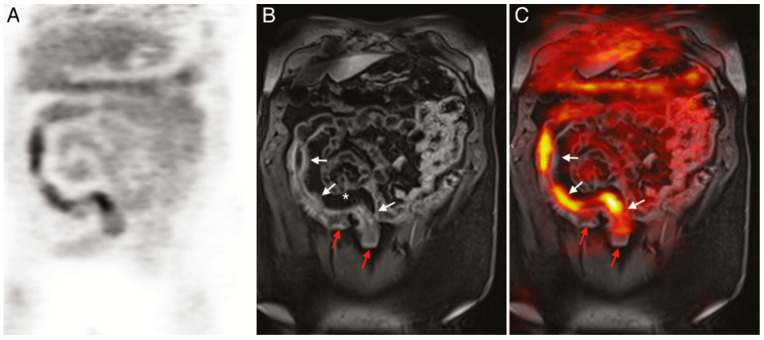
(**A**) ^18^F-FDG PET image of human patient with Crohn’s disease. (**B**) T1-weighted magnetic resonance imaging (MRI) image of the same patient. (**C**) Merged PET/MRI. White arrows indicate locations of acute inflammation while red arrows highlight damage resulting from earlier disease action. The asterisk (*) shows a site of proliferation of fibrofatty compounds in the mesentery. SUVmax of ^18^F-FDG 5.6–9.2 vs. SUVmax of background bowel 1.5–2.8 [147].

PET imaging is currently the only clinically approved molecular imaging approach for GI inflammation [143,144]. Specifically, ^18^F-FDG PET is used to measure the extent and magnitude of GI inflammation, indicating areas of low or high inflammation based on metabolic differences throughout the GI tract. The high metabolic need of inflamed tissue alongside the increased presence and activity of immune cells, such as leukocytes, results in increased glucose metabolism at sites of inflammation [145]. Differences in ^18^F-FDG consumption highlight areas of increased inflammation while contrasting against normal healthy tissue. PET alone offers limited spatial resolution despite its potential for high contrast imaging. Additionally, the uptake of ^18^F-FDG occurs in off-target sites, resulting in high background signal. As such, PET is frequently paired with either CT or MRI imaging to better monitor disease status and accurately assess disease location, as shown in (Figure 5) [146,147,148].

Current molecular imaging techniques prove mostly effective for verifying the extent and magnitude of GI inflammation. Preclinically, there has been investigation into the manipulation of contrast agents for the molecular imaging of GI inflammation. Wang et al. quantified inflammation in acute colitis mouse models using ultrasound with a P- and E-selectin targeted contrast agent and ^18^F-FDG-PET/CT. Similar results were obtained with both modalities [149]. P- and E-selectin are overexpressed on endothelial cells at sites of active inflammation, suggesting the future utility of this work in inflammatory GI disorders. While not practiced in the clinic at this time, immuno-PET techniques use radiolabeled proteins to target the upregulated immune cell presence or biochemical activity around inflamed tissues [150,151]. For example, antibody fragments targeting mouse CD4 cells, which are increasingly present at sites of GI inflammation, indicated the location and intensity of colorectal inflammation in mouse models [152]. Another modality undergoing preclinical assessment for the imaging of inflammation is multispectral optoacoustic tomography (MSOT). MSOT permits accurate, non-invasive imaging of the molecular characteristics of the disease through the visualization of exogenous or endogenous contrast agents [153,154]. Preclinical MSOT analysis has been shown to accurately detect in vivo colitis through measuring hypervascularity, which is common in inflamed tissue, and oxyhemoglobin levels in inoculated mouse models [155]. Alongside imaging modalities, new molecular targets are being investigated for improved diagnostic capabilities. α_4_β_7_ integrin is currently under investigation to determine whether it has the potential to increase the accuracy of IBD imaging. This is based on the increased presence of α_4_β_7_ integrin on the activated lymphocytes found in inflamed tissue [156,157]. Endothelial growth factor receptor (EGFR) may be another target for imaging given its overexpression in inflamed and malignant cells. One study demonstrated the ability of radiolabeled anti-EGFR antibody fragments to successfully detect sites of IBD in mouse models, presenting greater target specificity and signal intensity relative to ^18^F-FDG [158]. As new markers, probes, and imaging modalities are developed or found, accuracy in imaging diagnoses and tracking of GI inflammation is sure to improve.

## 3. Cancer

**Figure 6:**
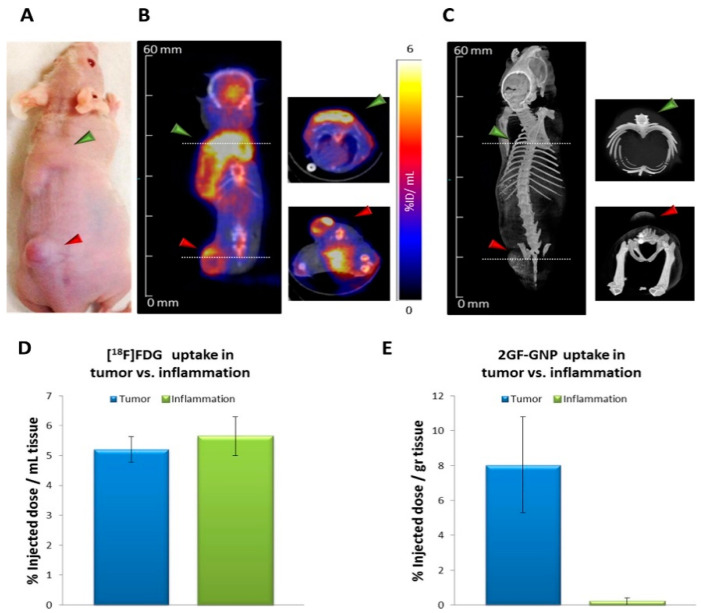
(**A**) Green arrowheads indicate the location of inflammation; red arrowheads indicate the location of A431 tumors. Images are taken after glucose-functionalized gold nanoparticles (2GF-GNP) injection. (**B**) ^18^F-FDG-PET/CT slice images of a representative mouse at 40–60 min post-injection. (**C**) CT surface-rendered images of the same mouse at 3.5 h post IV injection of 2GF-GNP. Quantification of ^18^F-FDG uptake and 2GF-GNP is shown in figures (**D**,**E**), respectively. ^18^F-FDG cannot differentiate between inflammatory lesions and tumor, while 2GF-GNP can [161].

The relationship between inflammation, infection, the immune system, and cancer is complex and still under investigation. As tumor cells proliferate, they secrete many cytokines and chemokines, which recruit leukocytes, often causing an inflammatory response. These leukocytes, such as tumor-associated macrophages (TAMs), have a key role in the development of the tumor microenvironment [159]. As previously mentioned, ^18^F-FDG PET imaging is commonly used to visualize inflammation and cancer through increased glucose metabolism [160]. This can be problematic when trying to differentiate active cancers from inflammatory lesions, since both cancer and inflammation have increased perfusion and metabolic activity. CT imaging of glucose-functionalized gold nanoparticles (GF-GNPs) was used in preclinical mouse models to differentiate cancer and inflammation based on differences the vasculature (Figure 6). [161]. Similarly, enhanced MRI imaging of ultrasmall superparamagnetic iron oxide particles (USPIO) was able to differentiate between inflammatory lesions and tumors [162]. Recent advances in multispectral optoacoustic tomography (MSOT) provide a single imaging modality that is capable of differentiating cancer from solely inflammatory lesions by imaging multiple biomarkers simultaneously. Many different inflammatory biomarkers are described in this paper; the labeling of these markers with an NIR-sensitive fluorophore will allow for visualization with MSOT. The development of NIR-sensitive, tumor-targeted imaging probes is currently a main focus of MSOT research [112,163,164,165]. MSOT can differentiate between multiple NIR-sensitive agents through the spectral unmixing of unique spectral shapes. This suggests that as long as inflammatory and tumor targeted imaging agents are spectrally distinct, then it will be feasible to visualize cancer and inflammation simultaneously. The ability to identify a small nidus of cancer in the setting of larger inflammation has significant potential to result in earlier stage diagnoses for patients with pancreas or colorectal cancers in the setting of pancreatitis or inflammatory bowel disease.

## 4. Imaging of Immunotherapy and Cellular Therapy

Therapeutics in malignancy have recently undergone a paradigm shift, with movement from classic chemotherapeutics focused on the interruption of growth with toxic metabolites to medications and cellular therapeutics created to upregulate and/or direct host immune systems. The use of checkpoint inhibitors that activate and enhance T-cell function is extensively used in both solid tumors and hematologic areas. Recently, cellular therapeutics have also been approved for the direction of modified T cells to receptor targets in both leukemia and lymphoma. With this shift of therapy, the diagnostic platforms of CT and FDG-PET typically employed in cancer imaging are no longer capable of clearly differentiating an immune response from malignancy progression. For this reason, newer imaging agents are being studied to track and monitor disease and immune response in an upregulated immune system.

Recent efforts to improve diagnostics in this arena have focused on labeling T cells with PET probes to effectively track and identify effects of T-cell activation in cellular therapy and graft vs. host disease (GVHD). In mouse models, FLT-PET has been used to identify proliferation in the gut, correlating with immune recognition and response, in the setting of graft versus host disease [166]. Additionally, human T cells with anti-melanoma T-cell receptor have been transduced with F-L-MAU plus hdCKEmut PET probes to track and monitor response to melanoma lesions [167]. HSV1-TK transduced lymphocytes and CD19 CAR T cells with truncated epithelial growth factor receptors have been used to provide a platform for both the imaging and tracking of cellular response, as well as the incorporation of suicide genes for safety in the setting of severe T cell immunologic response or graft vs. host disease [168,169]. Recently, a rodent model has adopted the use of ^89^ZrDFO-Inducible T-Cell COStimulator (ICOS)-monoclonal antibody (ICOS-ImmunoPET), taking advantage of elevated ICOS in activated CAR-T cells and thereby tracking response and localization [170]. Multiple studies using novel PET isotopes incorporated with either MRI or CT are under study to improve diagnostic accuracy in GVHD, cellular therapy, and immune-based therapeutics. (NCT03633955 FLT-CT in immunotherapy, F-18 ARA-G PET (NCT03367962, NCT03546556 FLT/MRI, NCT03802123 89Zr-Df-AB22M2C PET/CT CD8 TIL in solid tumor response). As therapeutics in cellular therapy advance, the allogeneic and immunologic sphere of cancer care expand into both hematologic and solid tumor areas, the capability to monitor, track, and quantitatively measure upregulated cellular components will be necessary, which will require the use of molecular imaging. In addition, future studies will also require molecular imaging to track the potential toxicity and early indications of efficacy.

## 5. Image Analysis of Inflammatory Disease

While this review focuses on the molecular imaging of inflammation for the diagnosis of inflammatory disease, there are many other methods for imaging inflammatory disease. Computer-assisted detection, segmentation, and classification of inflammatory tissues in the body have been the subject of several studies. These studies obtain images through various modalities (e.g., video endoscopy, infrared, or thermal imaging, etc.), and develop algorithms to recognize the characteristics of inflammation. While initial studies relied on classical machine learning and image analysis techniques, recent work heavily uses deep learning techniques. The traditional machine learning techniques can be characterized based on the extracted features and the type of classifier. The feature extraction techniques include gray-level co-occurrence matrix (GLCM), gray-level run-length (GLRL), speeded-up robust feature extraction (SURF), and dual-tree m-band wavelet transform (DTMBWT) algorithms [171,172,173,174,175,176]. The classifiers include support vector machine (SVM), k-nearest neighbor (K-NN), Random Forest (ensemble classifiers on three ensemble algorithms: bagging, Adaboost, and random subspace), and fuzzy c-means clustering (FCM) [177].

Recent advances in computational infrastructures and the availability of large datasets with ground truth have accelerated deep learning-based techniques. Their application to inflammation analysis from medical images has started. These studies rely on Convolutional Neural Networks (CNN) and CNN-based transfer learning methods (e.g., Residual network-50 (ResNet-50) and ResNet-34 [178], VGG-16 [179], and Inception-V3 [180], InceptionResnetV2 [181], and NASnet (mobile) [182]). Transfer learning methods employ pre-trained networks and retrain them with new domain-specific images but require a smaller number of images.

**Figure 7:**
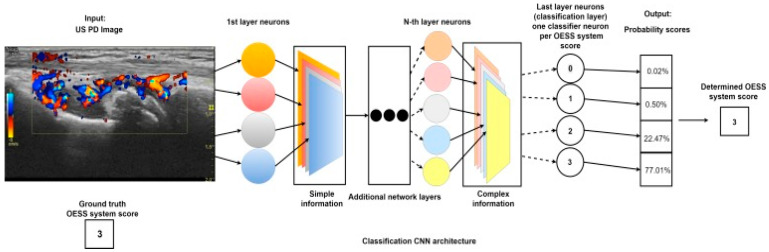
Schematic of the convolutional neural network that scores disease severity of RA based on an ultrasound color Doppler image of the wrist. Synovitis activity is evaluated and scored from 0–3, based on the OMERACT-EULAR Synovitis Scoring (OESS) System. After passing through each layer of the neural network, the classification neuron will map the resulting information to probability scores for each OESS score. The score with the highest probability is assigned to the US image [189].

These methods were applied to various organs and inflammatory diseases, particularly rheumatoid arthritis (RA), which is the most common inflammatory and systemic connective tissue disease [183,184]. Some of the RA-related studies focus on hand images captured with different imaging modalities, such as infrared thermography sensor [185], thermal image [172], and digital anterior–posterior radiographs of hand images [186,187]. Some of these studies (e.g., [172,185]) use traditional image processing and machine learning algorithms such as thresholding, dilation, erosion, depth-first search (DFS), gray-level co-occurrence matrix (GLCM), and k-means. Other studies (e.g., [186,187]) use a CNN-based approach to segment and detect the RA regions. A review paper summarizes machine learning studies in rheumatic diseases [188]. Some studies go beyond detection and segmentation to scoring severity (Figure 7) [173,189]. Computerized analysis of inflammation was also applied to other inflammation diseases: paranasal sinus [190,191], chronic obstructive pulmonary disease (COPD) [177,192,193,194], celiac disease (CD) [195,196,197,198], inflammatory gastrointestinal lesions [176,199,200], varicose veins [201], myocarditis [202,203], and inflammatory brain abnormalities.

Table 2 further demonstrates the broad spectrum of the imaging modalities and image analysis techniques employed to detect, segment, or classify inflammatory diseases. These studies report the performance of their algorithms with commonly used metrics of precision, accuracy, recall, specificity, F1 score, loss, the area under the curve (AUC), true positive rate (TPR), positive predictive value (PPV), and Dice coefficient. The performances vary from study to study. To give some idea of these performance values, the range of the Dice coefficient for sinusitis segmentation is 86–97%, the accuracy is 78% to 92% [190,191,204]. For COPD and lung disease inflammation, and the accuracy ranges from 61% to 95% [177,192,193,195,205]. For CD, the accuracy ranges from 79% to 97%, while the sensitivity and specificity vary from 83% to 100% and 96% to 100%, respectively [195,196,197,198].

Many reported studies need larger training datasets to better characterize the bias among different imaging modalities and to improve their performance and generalizability because of the variability in datasets. These studies also highlight the need for stronger clinical significance. For example, in a recent study of sinusitis, CNN scores were correlated with Lund–MacKay (LM) scores, which is the clinical visual score [190,214,215], while evaluating clinical significance was left as future work. Some studies also point out the need for further algorithmic development, such as the need for reliable methods for separating individual sinus cavities [190]. For COPD and inflammatory lung disease, microscopic image scoring algorithm accuracies are reported to be similar to those of pathologists; however, both computer algorithms and pathologists struggle in discriminating red blood cells from inflammatory cells when the staining was very dark [194]. For the CD, IBD, and inflammatory gastrointestinal lesion studies, it is often difficult to select the informative parts of the endoscopy and colonoscopy videos because of contributions from out-of-focus areas and image quality problems. For these studies, future work must be done to associate clinical findings with endoscopy results to have a fully automated system. Multiple sclerosis (MS) is a chronic inflammatory diseases of the brain which often requires segmentation of brain MRI images. The 3D patch-wise CNN approach has been used to segment the brain [211,212,213], but a large spatial variability makes the segmentation challenging. In addition, using 3D CNN needs more volumetric data (weights) to prevent overfitting.

## 6. Conclusions

Inflammatory diseases are extremely common and have high morbidity and mortality rates in severe cases. Early identification of molecular characteristics is the best chance at stopping irreversible damage. There is a demand for a non-invasive and highly specific way to image the pathophysiology of these diseases. As the immune system plays a huge role in inflammatory disease, immune cells and corresponding inflammatory cytokines are the primary targets in the molecular imaging of inflammatory disease. Advances in molecular imaging enable earlier detection through specific biomarkers that may be present before the onset of symptoms, leading to better patient care.

## Figures and Tables

**Table 1:** Summary of the molecular targets and tracers used to identify inflammatory disease that are discussed in this review.

Disease	Target	Tracer	Inflammatory Component	Source
Cardiovascular Disease	Glucose Metabolism	^18^F-Flourodeoxyglucose (FDG)	Activated macrophage accumulation	[41,42,43,44]
	Translocator protein (TSPO) receptors	^11^C- PK11195 ^18^F-GE-180	Overexpressed on activated macrophages	[51,52,53,54]
	Somatostatin receptor subtype-2 (SSR-2)	^68^Ga-DOTATATE/ ^64^Cu-DOTATATE	Overexpressed on activated macrophages	[56,57]
	Chemokine receptor 4	^68^Ga-pentixafor	Overexpressed on activated macrophages	[59,60,61]
	Hypoxia	^18^F-fluoromisonidazole (FMISO	Activated macrophage accumulation → inflammation and thickening of the vessel wall → decreased oxygen diffusion efficiency → Hypoxia	[64]
		^18^F-EF5		[65]
Rheumatoid Arthritis	Glucose metabolism	^18^F-Flourodeoxyglucose (FDG)	Activated macrophage accumulation	[90,91,92]
	Folate receptor β (FRβ)	^18^F-Fluoro-PEG-folate ^111^In-folate conjugate	Overexpressed on activated macrophages within the synovial fluid	[93,95,96,97]
		NIR2-Folate		[98]
	E-selectin	^111^In-labeled anti-E-selectin MAb	Overexpressed on endothelial cells due to TNFα	[100]
		DyLight 750/anti-E-selectin Mab probe		[87]
		^99m^Tc-labelled anti-E-selectin FAb		[102]
	MMPs	^18^F-pyriminde-2,4,6,-triones	Elevated levels in synovial fluid correlate with inflammatory response	[104]
		NIR fluorescent MMP-3 specific chitosan nanoparticle		[103]
	CD20	^124^I-Rituximab ^89^Zr-Rituximab	Overexpressed on B lymphocytes as they accumulate in synovial fluid	[105,106]
	TNFα	^99m^Tc-Infliximab	Overexpressed in synovial fluid	[107,109]
	L-selectin/P-selectin	NIR Fluorescent Polyanionic dendritic polyglycerol sulfate (dPGS)	Movement of immune cells to the inflammatory location	[111,113]
COPD	Pulmonary perfusion	^99m^Tc-labeled macroaggregated albumin	Ventilation/Perfusion (V/Q) scintigraphy to regional inflammatory/airflow differences	[123,125]
	Pulmonary ventilation	^81m^Kr or ^133^Xe ^99m^Tc-labeled DTPA ^99m^Tc-labeled carbon particles (Technegas)		[125]
	Glucose metabolism	^18^F-Flourodeoxyglucose (FDG)	Activated macrophage accumulation	[130,131,132,133]
	Translocator protein (TSPO) receptors	^11^C-PK11195	Overexpressed on activated macrophages	[134]
	MMPs	^18^F-IPFP	Produced by active macrophages at the inflammatory location	[135]
		^99m^Tc-labeled RP805		[136]
Gastrointestinal	Glucose metabolism	^18^F-Flourodeoxyglucose (FDG)	Activated macrophage accumulation	[143,144]
	CXCL8 receptor	^99m^Tc-CXCL8	Overexpression on activated neutrophils	[150]
	Interleukin 1 β	^89^Zr-lα-IL-1β	Secreted by immune cells indicating an inflammatory response	[151]
	CD11b	^89^Zr-α-CD11b	Pan-myeloid innate immune marker	[151]
	CD4	^89^Zr-GK1.5 cys diabody (cDb)	CD4 positive T-Cells characterize IBD inflammatory response	[152]
	EGFR	^64^Cu-Cetuximab fragment-DOTA	Overexpression in inflammatory cells	[158]

**Table 2:** Inflammatory diseases, imaging modalities, and image analysis techniques of the studies stated in the reference column.

Inflammatory Disease	Imaging Modalities	Image Analysis Techniques	Source
Rheumatoid arthritis (RA)	CT, Thermal Image	GLCM, KNN, Random Forest, DFS, K-Means Clustering	[172,173,185,186,187,188,189,206]
Paranasal sinus Chronic rhinosinusitis (CRI)	CT, Radiography Images	CNN-Based Segmentation, CNN-Based Transfer Learning	[190,191,204]
Chronic Obstructive Pulmonary Disease, Detecting Lung Disease, Fibrotic and inflammatory Lung Disease	CT, X-Ray Images Microscopy Images (Whole Slide Images)	GLCM, CNN, FCM, CNN-Based Transfer Learning	[177,192,193,194,205]
Celiac Disease (CD)	Endoscopy Images H&E Duodenal Biopsy Images	CNN-Based Transfer Learning (Alexnet, VGG Nets, Resnet) SVM, Bayesian	[195,196,197,198]
Inflammatory Bowel Disease (IBD) Inflammatory Gastrointestinal Lesion	Histology and Endoscopy Images Colonoscopy Images	CNN, SURF, CNN-Based Transfer Learning (Resnet-152, Inception-Resnet-V2)	[173,199,200,207]
Varicose Vein	Multi-Scale Image	CNN	[201]
Myocarditis	Cardiac MRI (CMR)	CNN, K-Means Clustering	[202,203]
Inflammatory Brain Abnormalities MS Segmentation	H&E Stain Image Magnetic Resonance Imaging (MRI)	R-CNN, DTMBWT, GLCM, GLRL, SVM, KNN, Random Forest	[208,209,210,211,212,213]

## Abbreviations

computed tomography: CT; magnetic resonance imaging: MRI; positron emission tomography: PET; ultrasound: US; superparamagnetic iron oxide nanoparticles: SPIONs; ultrasmall superparamagnetic iron oxide particles: USPIO; chemical exchange saturation transfer: CEST; cardiovascular disease: CVD; rheumatoid arthritis: RA; power Doppler ultrasound: PDUS; chronic obstructive pulmonary disease: COPD; diffusion-weighted MRI: DWI-MRI; gastrointestinal: GI; antibodies to citrullinated protein antigens: ACPAs; rheumatoid factor: RF; indocyanine green: ICG; inflammatory bowel disease: IBD; gray-level co-occurrence matrix: GLCM; gray-level run-length: GLRL; speeded-up robust feature extraction: SURF; and dual-tree m-band wavelet transform: DTMBWT; algorithms, support vector machine: SVM; k-nearest neighbor: K-NN; fuzzy c-means clustering: FCM.
